# Applications and Comparison of Dimensionality Reduction Methods for Microbiome Data

**DOI:** 10.3389/fbinf.2022.821861

**Published:** 2022-02-24

**Authors:** George Armstrong, Gibraan Rahman, Cameron Martino, Daniel McDonald, Antonio Gonzalez, Gal Mishne, Rob Knight

**Affiliations:** ^1^ Department of Pediatrics, School of Medicine, University of California, San Diego, La Jolla, CA, United States; ^2^ Bioinformatics and Systems Biology Program, University of California, San Diego, La Jolla, CA, United States; ^3^ Center for Microbiome Innovation, Jacobs School of Engineering, University of California, San Diego, La Jolla, CA, United States; ^4^ Halıcıoğlu Data Science Institute, University of California, San Diego, La Jolla, CA, United States; ^5^ Department of Computer Science and Engineering, University of California, San Diego, La Jolla, CA, United States; ^6^ Department of Bioengineering, University of California, San Diego, La Jolla, CA, United States

**Keywords:** microbiome, dimensionality reduction, ordination, sequencing data, non-linear embeddings

## Abstract

Dimensionality reduction techniques are a key component of most microbiome studies, providing both the ability to tractably visualize complex microbiome datasets and the starting point for additional, more formal, statistical analyses. In this review, we discuss the motivation for applying dimensionality reduction techniques, the special characteristics of microbiome data such as sparsity and compositionality that make this difficult, the different categories of strategies that are available for dimensionality reduction, and examples from the literature of how they have been successfully applied (together with pitfalls to avoid). We conclude by describing the need for further development in the field, in particular combining the power of phylogenetic analysis with the ability to handle sparsity, compositionality, and non-normality, as well as discussing current techniques that should be applied more widely in future analyses.

## Introduction: What Is Dimensionality Reduction and Why do We do It?

To a first approximation, life on Earth consists of complex microbial communities, with “familiar” multicellular organisms such as plants and animals being rounding errors in terms of cell count and biomass. The genetic repertoire of such a community is called a “microbiome” (Turnbaugh et al., 2007), although the term “microbiome” is often also loosely applied to the collection of microbes that make up the community. In either sense, microbiomes are typically incredibly complex, containing vast numbers of species and genes, and how samples relate, even in well-studied contexts, are not predetermined. For example, in the Earth Microbiome Project (EMP) (Thompson et al., 2017) and the work leading up to it (Lozupone and Knight, 2007; Ley et al., 2008; Caporaso et al., 2011), an ontology constructed from the microbe’s perspective based on community similarities and differences revealed many surprises, such as a deep separation between free-living and host-associated samples, and between saline and non-saline samples. Accordingly, to truly understand the microbial perspective, we must get acquainted with the structure of the data in human-interpretable formats. This is especially important when we need to separate new biological discoveries from technical artifacts, such as distinguishing clusters related to different habitats on the human body from artifacts caused by different sequencing methodologies such as PCR primers (The Human Microbiome Project Consortium, 2012).

**FIGURE 1:**
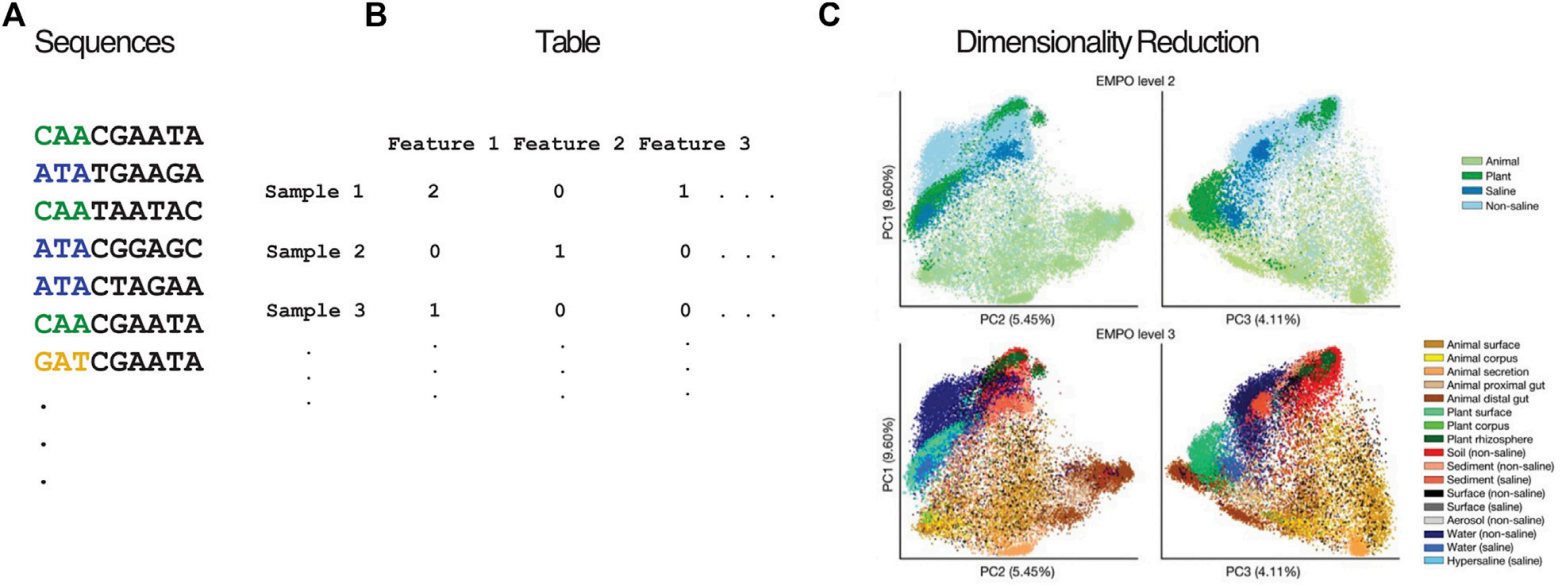
Overview of dimensionality reduction pipeline. Nucleotide sequences **(A)** from a biological experiment are organized in a feature table **(B)** containing the abundance of each feature (e.g., OTU, ASV, MAG) in each sample. **(C)** Beta diversity plots showing unweighted UniFrac coordinates of EMP annotated by EMPO levels 2 and 3. **(C)** is a derivative of Figure 2C from “A communal catalogue reveals Earth’s multiscale microbial diversity” by Thompson et al. (2017) used under CC BY 4.0.

When microbiome sequencing data (Figure 1A) are arranged into count tables (Figure 1B), such as those that count 16S amplicon sequence variants (ASVs) or the microbial genes present in a sample, the number of features being counted across all of the samples often vastly outnumbers the number of samples observed. This phenomenon of having many features, and particularly having far more features than samples, is a hallmark of high-dimensionality. For example, the EMP (Thompson et al., 2017) contained 23,828 samples and represented 307,572 ASVs, where each of these ASVs is considered a dimension of the resulting count table. This degree of high feature dimensionality creates difficulties for interpreting data and calculating meaningful statistics, since humans cannot visualize more than 3 dimensions, many of the features are noisy or redundant, the number of hypotheses that explain the data is far greater than the number of observations, and the number of features can cause run-time issues for downstream analysis. These are all common consequences of the “curse of dimensionality”. Dimensionality reduction transforms a high-dimensional dataset into a representation with fewer dimensions, while retaining the key relationships among samples from the full dataset, making analysis tractable. Accordingly, dimensionality reduction is a core step in microbiome analyses, both for creating human-understandable visualizations of the data and as the basis for further analysis. The EMP used dimensionality reduction to produce plots of the 23,828 samples using 3 coordinates (in contrast to the 307,572 ASVs) that demonstrate the large difference between host-associated and non-host-associated microbiomes, and between saline and non-saline free-living microbiomes (Figure 1C). These differences in microbial communities were subsequently statistically validated. This example is particularly salient because it shows the value of preserving the structure of the data while using much less information to represent it. Owing to its importance, dimensionality reduction methods are included in many analysis packages, including QIIME 2 (Bolyen et al., 2019), mothur (Schloss et al., 2009), and phyloseq (McMurdie and Holmes, 2013), as well as online software such as Qiita (Gonzalez et al., 2018) and MG-RAST (Keegan et al., 2016).

In this review, we describe how the characteristics of microbiome data complicate dimensionality reduction. We then discuss common strategies for dimensionality reduction (Table 1), examining in detail whether and how they address each of the aspects that, in conjunction, confound microbiome analysis. Tried-and-true techniques, although useful, often have conceptual and practical problems that limit their utility in the microbiome, due to the inability to handle the data’s most salient traits simultaneously (Table 2). In this light, we then focus on examples of how dimensionality reduction techniques have been used in the literature, highlighting biological findings that have been revealed by each, while also discussing what may have been obscured. We then discuss common artifacts of widely used dimensionality reduction techniques, including specific pitfalls that users of these techniques must avoid in order to draw conclusions that are robust, reproducible, and well-supported by their data. We end with guidance on how dimensionality reduction should be used responsibly by practitioners in the field, and with an outlook describing how additional techniques that are seldom used today might provide valuable advances.

**TABLE 1:** Common characteristics of strategies for dimensionality reduction address different aspects of the data.

Table 1		
Term	Definition	
Compositionally aware	Transforms data to account for non-independence of features in sequence count data	
Pseudo-counts or imputation	Requires no/minimal zeroes in the feature table due to numerical issues (such as logarithm transform being undefined on zeroes)	
Able to incorporate phylogeny	Method is calculated with awareness of how each sampled microbial community is evolutionarily represented relative to other samples	
Operates on beta-diversity dissimilarities	Dimensionality reduction step is performed on pairwise dissimilarities (arbitrary metric) between samples, rather than the feature table itself	
Linear	Lower dimensional coordinates are computed via linear transform of features	
Repeated measures	Subjects are sampled multiple times. Commonly sampled longitudinally	
Feature relationships are interpretable	The method indicates the relevance of input microbial features with regard to its output coordinates	
Supervised component	Method takes explanatory sample variables as an additional input	

**TABLE 2:** Dimensionality reduction methods each have their own characteristics. x indicates that the characteristic applies to the method. Examples of software capable of performing each method are included in the last column.

Table 2									
	Compositionally aware	Avoids pseud-counts or imputation	Able to incorporate phylogeny	Operates on beta-diversity dissimilarities	Linear	Repeated measures	Feature relationships are interpretable	Supervised component	Software
PCoA	—	x	x	x	x	—	—	—	QIIME 2, CRAN phyloseq, mothur
PCA	—	x	—	—	x	—	x	—	scikit-learn, R built-in, mothur
UMAP	—	x	x	x	—	—	—	—	umap-learn, CRAN umap, QIIME 2
t-SNE	—	x	x	x	—	—	—	—	scikit-learn, CRAN tsne
nMDS	—	x	x	x	—	—	—	—	scikit-learn, CRAN vegan, mothur, CRAN phyloseq
CCA	—	—	—	—	x	—	x	x	scikit-bio, CRAN vegan, CRAN phyloseq
PLS-DA	—	—	—	—	x	—	x	x	CRAN mixOmics
Aitchison PCA	x	—	—	—	x	—	x	—	scikit-bio, QIIME 2
RPCA	x	x	—	—	x	—	x	—	gemelli, QIIME 2, vegan
CTF	x	x	—	—	x	x	x	—	gemelli, QIIME 2

### Specific Features of Microbiome Data That Complicate Dimensionality Reduction

“Microbiome data” most often refers to sequencing results from two primary methodologies. The first class of microbiome sequencing is known as “amplicon sequencing” where a specific gene or region of a gene is targeted in each sample. 16S, 18S, and ITS sequencing approaches all fall under this class of methods. Variants of the targeted nucleotide sequences are used as a proxy for discrete microbial taxa. These unique sequences can be clustered by sequence similarity into “operational taxonomic units” (OTUs) or used by themselves as individual units after denoisers, such as DADA2 and Deblur, resolve the individual sequence variants from error-prone sequences (Callahan et al., 2017; Amir et al., 2017). These filtered sequences are often called amplicon sequence variants (ASVs) (Callahan et al., 2017) or sub-OTUs (sOTUs). The second class of microbiome sequencing is shotgun or whole metagenome sequencing. In this method, the DNA from a sample is collected and sequenced broadly. The reads are then mapped to a reference database to determine the corresponding units, which can range from taxonomic identities to gene families or genes from a specific reference genome or metagenome-assembled genomes (MAG).

The result of these sequence analysis pipelines is typically a “feature table” that counts the microbial “units” or features (OTU, ASV, MAG, etc., (Figure 1B)) associated with each sample. Additionally, information about the relationship between features, such as taxonomic identity or gene family, can optionally be used to “collapse” the feature table to a lower resolution sum of its units. At this point, the data are generally ready to pursue exploratory analysis with dimensionality reduction.

**FIGURE 2:**
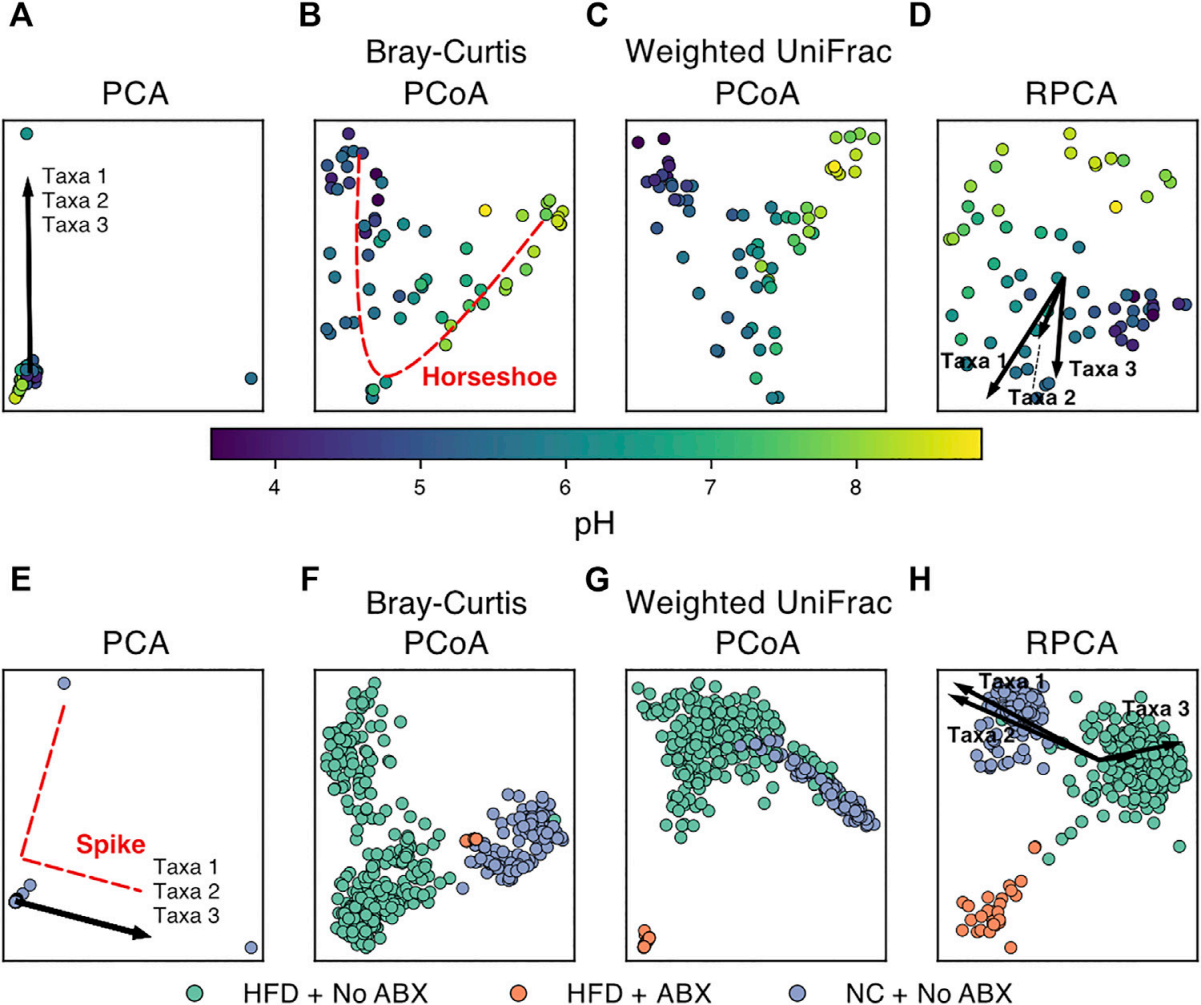
Examples of dimensionality reduction techniques applied to publicly available microbiome data. (Top) Beta-diversity plots of soil samples colored by pH from (Lauber et al., 2009). (Bottom) Beta-diversity plots of murine fecal samples colored by diet and antibiotics usage from (Shalapour et al., 2017). (HFD = high-fat diet, NC = normal chow, ABX = antibiotics). PCA plots **(A,E)** show extremely high sample overlap due to outliers and characteristic “spike” artifacts. The top three taxa driving variation also overlap as shown by arrow superposition. **(B)** “Horseshoe” pattern emerges for samples following ecological gradients such as pH. RPCA plots **(D,H)** show the top three taxa driving separation of groups. **(F)** and **(G)** show strong overlap of HFD + ABX samples resolved by **(H)**.

However, there are several features common to microbiome data that can make standard dimensionality reduction techniques difficult to apply or to interpret. Each method must therefore handle each of these key issues or be benchmarked carefully to determine that these issues do not strongly affect the results in ways that are problematic for biological interpretation. We demonstrate various dimension reduction techniques on two datasets: Lauber et al., 2009 (Figures 2A–D) and Shalapour et al., 2017 (Figures 2E–H) looking at soil pH and antibiotic-diet axis respectively.

*High dimensionality*. In this context, “dimensionality” refers to the number of features in a feature table. Microbiome data typically have far more features than samples. Across studies ranging from tens of samples to tens of thousands of samples, the number of features for taxonomic data typically exceeds the number of samples by 20-fold or more. With gene-oriented data, the number of genes represented in a metagenomic study typically exceeds samples by several orders of magnitude. This can lead many statistical methods to overfit or to produce artifactual results.

*Sparsity.* Most microbes are not found in most samples, even of the same biospecimen type, for example, most human stool specimens from the same population have relatively low shared taxa (Allaband et al., 2019). As a result, a feature table containing counts of each microbe in each sample often has many zeros corresponding to unobserved microbes. Most 16S microbiome datasets do not have even as many as 10% of the possible entries observed in most of the specimens. Feature tables with this over-abundance of unobserved counts are said to be “sparse”, posing problems for statistical analysis. Moreover, the proportion of observed values tends to decrease as additional samples are sequenced, often leading to tables with density well below 1% (Hamady and Knight, 2009; McDonald et al., 2012).

*Compositionality.* In any high-throughput sequencing experiment, we impose an implicit limitation and randomness to the number of reads from a given sample due to many factors, including the random sub-sampling occurring in the process of collecting samples as well as uncontrolled variation in how efficiently each sample is amplified and incorporated into molecular libraries for sequencing. This limitation, termed “compositionality”, should always be kept in mind when performing any microbiome analysis on abundance data (Gloor et al., 2017). The total number of sequences per sample can affect the distances between samples (Weiss et al., 2017). Strategies such as rarefaction and relative abundance normalization are common for normalizing differences in sequencing depth. However, the relative amount of one feature in the sample is not independent from the counts of the other features. A difference in just one feature of the original sample can induce an observation that many other features are also changing (Morton et al., 2019) and neither rarefaction nor relative abundance sampling solve this issue. Due to this effect, many dimensionality reduction methods, such as PCA, will emphasize false correlations in the data.

*Repeated measures.* One of the most challenging experimental aspects to account for in dimensionality reduction is repeated measures data, e.g., multiple timepoints from the same subject where the variation between subjects may be greater than the variation between timepoints (Wu et al., 2011). In the context of dimensionality reduction, subjects or sites with multiple samples represented (such as in longitudinal studies or replicate analysis) provide an additional source of variation that can inhibit interpretation of the experimental effect of interest; the samples from a single subject can be highly correlated, resulting in between-subject differences dominating the ordination [e.g., (Song et al., 2016)].

*Feature interpretation.* Analysis of high-dimensional microbiome data is often motivated to find microbial biomarkers associated with observed differences in sample communities (Fedarko et al., 2020). This line of inquiry is of interest for diagnosis and/or prognosis of disease status, dysbiosis, and a host of other biological questions. Although this task is often addressed with differential abundance methods, those methods make specific statistical assumptions and may not correspond to the group separation observed in an exploratory analysis performed with any dimensionality reduction method (Lin and Peddada, 2020). Thus, methods that offer a quantitative justification of their representation in terms of the microbial features are often desirable. However, methods with feature importance that are not specifically designed for the microbiome often fail to account for compositionality, which can include many false positives due to the induced correlation of features, and sparsity, where important but infrequently observed features will not be detected (false negatives).

*Complex patterns.* Microbiome data are often assumed to contain clusters or gradients (Kuczynski et al., 2010). For example, multiple samples swabbed from one’s own keyboard are more likely to be similar to each other than samples from another individual’s keyboard (Fierer et al., 2010), and the microbial composition of soils is expected to vary continuously with soil pH (Lauber et al., 2009). However, with larger and larger datasets with many covariates and metadata on these being collected, more complex patterns can be detected (Debelius et al., 2016), such as grouping by both biological and technical factors in the case of the Human Microbiome Project (The Human Microbiome Project Consortium, 2012). Furthermore, many conventional dimensionality reduction methods, such as principal component analysis (PCA), assume the data lie in a linear subspace, and this assumption is violated by microbiome data (Ginter and Thorndike, 1979; Greig-Smith, 1980; Potvin and Roff, 1993; Tabachnick and Fidell, 2013).

### Strategies for Dimensionality Reduction in the Microbiome

The problems that complicate dimensionality reduction in microbiome data are scattered throughout the analysis pipeline. Difficulties can arise immediately from the raw sequence count data. Many can be corrected before the dimensionality reduction step, with careful preprocessing, though this can raise other issues. Furthermore, beta-diversity analysis, which seeks to quantify the pairwise differences in microbial communities among all samples with dissimilarity metrics (tailored to microbiome data), is often helpful for addressing many of the aforementioned circumstances (Pielou, 1966). Algorithms that are able to incorporate these metrics are particularly valuable, and this can be done in a variety of ways. Finally, additional constraints can be placed on dimensionality reduction algorithms to account for study design or provide additional information about the correspondence between the features and the reduced dimensions. In this section, we discuss each of these strategies in depth.

*Compositionally Aware:* Comparisons between and among samples must consider how sampling and sequencing depth can affect projection into low-dimensional space. Traditionally, compositionality has been addressed using logarithmic transformations of feature ratios. Transformations such as the additive log-ratio (ALR), centered log-ratio (CLR), and isometric log-ratio (ILR) can convert abundance data to the space of real numbers such that analysis and interpretation are less skewed by false positives (Aitchison and Greenacre, 2002; Pawlowsky-Glahn and Buccianti, 2011). After transformation, the Euclidean distance can be taken directly on the log-ratio transformed data (referred to as Aitchison distance) (Aitchison and Greenacre, 2002). Dimensionality reduction methods that incorporate log-ratio transformations attempt to preserve high-dimensional dissimilarities while taking into account the latent non-independence of microbial counts.

*Pseudocounts and Imputation:* High-dimensional microbiome data is almost always plagued by problems of “sparsity”, or an overabundance of zeroes. The data transformations to address compositionality (as outlined above) are often based on logarithmic functions which are undefined at zero. The simplest solution is to add a small positive pseudocount to each entry of the feature table so that logarithmic functions can be applied. However, downstream analyses based on this approach are sensitive to the choice of pseudocount (Kumar et al., 2018) and there does not exist a standardized way to choose such a value. Other options include imputation of zeros (Martín-Fernández et al., 2003) through inference of the latent vector space. Fundamentally, zero handling is complicated by the inherent unknowability of the zero generating processes for each zero instance. In Silverman et al. (2020), they characterize the three different types of zero-generating processes (ZGP) as sampling, biological, and technical and demonstrate how the results of different zero-handling processes are affected by the (unknowable) mix of ZGPs in a given dataset. Recently Martino et al. (2019) introduced a version of the CLR transform that only computes the geometric mean on the non-zero components of a given sample. This avoids the problem of logarithms being undefined at 0 and thus dimensionality reduction through this method is robust to the high levels of sparsity in microbiome data.

*Incorporating Phylogeny:* Organisms identified using microbiome data can be related to one another through hierarchical structures that describe their evolutionary relationships. Typically, these structures take the form of either a taxonomy or a phylogeny. A taxonomy is a description of the organism relationships, generally derived subjectively using multiple biological criteria. A phylogeny, in contrast, is an inference of a tree, commonly with branch lengths, derived from quantitative algorithms that are typically applied to microbial, nucleic acid, or protein sequence data. Taxonomies have the advantage of being more directly interpretable because hierarchical structures correspond to a defined organization and classification pattern curated by experts in the field. However, these assignments and hierarchies are often putative and subject to change as more information about microbial taxa emerges. In contrast, phylogenies are derived from quantitative measures of sequence similarity from sample reads. These data structures are more easily incorporated into statistical analyses but often at the cost of less interpretability as the hierarchical structures do not necessarily map to pre-defined microbial relationships. These evolutionary relationships, particularly phylogenies, add information to microbiome analysis, because related organisms are more likely to exhibit similar phenotypes (although counterexamples do exist, especially closely related taxa such as *Escherichia* and *Shigella*, which are very similar genetically but produce different clinical phenotypes).

When comparing the similarity of pairs of microbial communities, it is possible to utilize these hierarchical structures, and derive a metric that computes a dissimilarity as a function of shared evolutionary history (Lozupone and Knight, 2005). Specifically, communities that are very similar will share most of their evolutionary history, whereas those that are very dissimilar will have relatively little in common. A popular form of phylogenetically-aware distances is the suite of UniFrac metrics, which includes both quantitative (Lozupone et al., 2007) and qualitative (Lozupone and Knight, 2005) forms. Numerous extensions to UniFrac have been developed (Chang et al., 2011; Chen et al., 2012), including variants that account explicitly for the compositional nature of microbiome data (Wong et al., 2016). Because these metrics all utilize not only exactly observed features, but also the relationships among features, they can better account for the sparsity of microbiome data which manifests at the tips of a phylogenetic tree (because most microbes are not observed in most environments). In contrast, a metric like the Euclidean distance is limited to only the information at the tips of these hierarchies, and, worse, assumes that all features at the tips are equally related to one another (so that in a tree consisting of a mouse, a rat, and a squid, there is no allowance for the fact that the two rodents are much more similar to each other than they are to the squid). Neither phylogenetic nor non-phylogenetic beta-diversity measures explicitly model differences in sequencing depth per sample, although these differences in depth can be standardized through rarefaction (Weiss et al., 2017).

*Operates on Generalized Beta-Diversity Matrix:* Many of the issues outlined above can be easily addressed at the sample dissimilarity level rather than directly through dimensionality reduction algorithms. A number of dissimilarity/distance metrics have been developed to account for factors such as phylogenetic data incorporation, compositionality, or sparsity that output a sample by sample matrix estimating high-dimensional dissimilarity. These dissimilarity matrices represent the overall community differences between pairwise samples calculated by a chosen beta-diversity metric. Dimensionality reduction methods that operate on arbitrary dissimilarity metrics are attractive options because the complex handling of the various feature table issues can be split into the choice of dissimilarity metric and the choice of dimensionality reduction algorithm. This adds a layer of flexibility for researchers to analyze their data depending on their needs. Methods based on multidimensional scaling approaches such as PCoA (Kruskal and Wish, 1978) and nMDS (Kruskal, 1964) attempt to preserve as much as possible the pairwise dissimilarities between subjects. Other methods such as t-distributed stochastic neighbor embedding (t-SNE) (van der Maaten and Hinton, 2008) and Uniform Manifold Approximation and Projection (UMAP) (McInnes et al., 2018) are non-linear dimensionality reduction techniques that aim to find a low-dimensional representation such that similar data points are placed closed together and dissimilar points are pushed apart. A caveat of these methods is that they can be very sensitive to the choice of dissimilarity used. Patterns that may appear from one measure of dissimilarity may not be as apparent in a different measure. As an example, phylogenetic metrics such as UniFrac may differ from non-phylogenetic metrics such as Bray-Curtis depending on the strength of phylogenetic contribution (Shankar et al., 2017). The choice of dissimilarity metric should therefore be considered carefully, as different dimensionality reduction techniques yield visually and statistically very different results on the same data (Kuczynski et al., 2011).

*Linear vs Non-Linear Methods:* Principal coordinates analysis (PCoA) and PCA are popular dimensionality reduction techniques that fall under the “linear” category. Linear techniques attempt to reduce or transform the data such that an approximation of the original data can be reconstructed by a weighted sum of the resulting coordinates. These methods typically involve computing decompositions/factorizations of the data that are highly computationally efficient and work well on data that is naturally linear. Various other techniques, such as robust Aitchison PCA (RPCA) (Martino et al., 2019), and nonnegative matrix factorization (NMF) (Lee and Seung, 1999) also fall under this class of techniques.

Other methods fall under the “non-linear” category, which perform more complex transformations that often excel at preserving different patterns that may not be linear. This category includes methods such as the non-metric multidimensional scaling (nMDS), t-SNE, and UMAP. These methods can more succinctly represent complex patterns, but possibly at the expense of additional computation. Furthermore, these models tend to have randomness (such as from initialization) and more hyperparameters that the output can be highly sensitive to, so it is usually necessary to run these algorithms multiple times to ensure the conclusions are reproducible. Other non-linear methods that have seen less frequent use in microbiome data (and bioinformatics generally) include kernel PCA (Scholkopf et al., 1999), locally linear embeddings (Roweis and Saul, 2000), Laplacian eigenmaps (Belkin and Niyogi, 2001), and ISOMAP (Tenenbaum et al., 2000).

Unlike its close, linear counterpart PCoA, nMDS performs the ordination onto a pre-specified number of dimensions and operates on the ranks of the dissimilarities, rather than the dissimilarities themselves. This rank-based approach can be beneficial for representing data that departs from the assumptions of linearity. Other non-linear methods, such as t-SNE and UMAP, also transform the data onto a pre-specified number of dimensions and operate by assuming the high-dimensional data follow a non-linear structure that can be represented with fewer dimensions.

*Repeated Measures:* If the biological variable of interest occurs at the subject level, repeated samples (such as through a longitudinal study design) can artificially inflate how tight a cluster appears in low-dimensional space. Dimensionality reduction methods for microbiome need to be designed for the purpose of handling this kind of data, with the intent to represent the relationships between explanatory variables while accounting for the inherent similarity between samples from the same subject. Methods to account for repeated measures can incorporate the relationship between individual samples and subjects by subject-aware decomposition of the data (Martino et al., 2021). There has also been discussion about incorporating prior sample relationship information into ordinations through Bayesian methods (Ren et al., 2017). Nevertheless, methods that incorporate repeated measures remain an underexplored area in dimensionality reduction literature.

*Feature Importance:* When the lower-dimensional representation of microbial communities shows separation between sample groups, a natural next question is what microbes or groups of microbes are driving such a separation. Dimensionality reduction methods that return a quantitative relationship between individual microbial features and the latent lower-dimensional space are a powerful class of methods that can demystify the construction of the lower-dimensional axes. However, certain methods that attempt to find high-dimensional patterns, such as non-linear methods, do not have an explicit interpretable correspondence between the output coordinates and the input features.

The most relevant category of methods for visualizing feature importance is the biplot ordination family of approaches. Biplots display both the samples and the driving variable vectors in reduced dimension space (Figures 2A,D,E,H). For example, PCA naturally quantifies the contribution of each microbe to the principal component axes through matrix factorization into linear combinations of features. RPCA modifies this approach to account for compositionality and sparsity while retaining interpretable feature loadings (Martino et al., 2019). Another set of ecologically motivated matrix factorization methods is the correspondence analysis (CA) family. The general CA method can be thought of as an implementation of PCA that operates on count data. It is also possible to explicitly incorporate sample metadata into these dimensionality reduction methods. Researchers are often interested in the explanatory power of their sample metadata (site, pH, subject, etc.). Certain dimensionality reduction methods can take as input both a feature table and a table of sample metadata to jointly estimate the low-dimensional representation of samples as well as the relative contribution of the provided metadata vectors. The general goal of these methods is to determine whether and/or which explanatory variables may be driving the differences in microbial communities among samples. Canonical correspondence analysis (CCA) is an extension of CA that incorporates sample variables of interest to determine which covariates are associated with the placement of samples and feature vectors in low-dimensional space (ter Braak, 1986). The results of CCA can be visualized as a “tri-plot” where samples are simultaneously visualized with the relative contribution of features and explanatory variables near related samples (Paliy and Shankar, 2016). Partial least squares discriminant analysis (PLS-DA) is a similar approach that uses only categorical sample metadata (classification) in the construction of lower-dimensional axes (Barker and Rayens, 2003; Ruiz-Perez et al., 2020). In each of these cases, the feature contributions can motivate subsequent statistical analysis of associations between sample metadata and specific microbial taxa.

### Uses of Dimensionality Reduction for Microbiome Data

Over the past decade, PCoA has seen an increase in use in microbiome analyses, and it is the primary ordination method for beta-diversity included by default in workflows such as QIIME2 (Bolyen et al., 2019). It is typically used for exploratory visualization, as it excels at rendering biologically relevant patterns, such as clusters and gradients (Kuczynski et al., 2010). When used as an exploratory tool, observed patterns are often followed with statistical analysis on the original feature tables or dissimilarity matrices (Galloway-Peña and Hanson, 2020), such as ANOSIM (Clarke and Ainsworth, 1993), PERMANOVA (aka Adonis) (Anderson, 2017), ANCOM (Mandal et al., 2015), or bioenv (Clarke and Ainsworth, 1993). It should also be noted that some of these statistical techniques use the full table or dissimilarity matrix, not the reduced dimension matrix as visualized (at least by default) and may therefore introduce incongruent results between the statistics and the visualization.

Exploratory visualizations have revealed microbial-associated patterns in applications ranging from host-associated gut microbiomes to soil, ocean, and other environmental microbiome contexts. For example, studies have applied PCoA to demonstrate differences between host groups, such as differences between humans’, chimpanzees’, and gorillas’ gut microbial taxa (Campbell et al., 2020), or the correspondence between human gut microbiomes and westernization (Yatsunenko et al., 2012; Campbell et al., 2020). Host microbiome-disease associations have also been identified using PCoA, such as in the case of colorectal cancer (Young et al., 2021) in humans and metritis in cows (Galvão et al., 2019). Uses also extend to host-environment relationships, such as demonstrating the differences between oyster digestive glands, oyster shells, and their surrounding soils (Arfken et al., 2017). The microbiome-shaping roles of environmental factors such as salinity in shaping free-living environments (Lozupone and Knight, 2007), pH in arctic soils (Malard et al., 2019) and depth in the ocean (Sunagawa et al., 2015) have also been elucidated with PCoA. In many of these cases, the PCoA visualizations demonstrated a separation between groups that was subsequently followed by statistical validation with PERMANOVA or ANOSIM.

In numerous other instances, PCoA has also been used to make claims that extend beyond exploratory group differences followed by statistical analysis. For example, Halfvarson et al. (2017) fit a plane to the healthy subjects in the first three coordinates of a PCoA and then used the distance to this plane to associate dissimilarities in the microbiome with the severity of irritable bowel disease (IBD) (Halfvarson et al., 2017); this approach has subsequently been replicated (Gonzalez et al., 2018). Others have used regression of participant and microbiome characteristics (e.g., age and alpha diversity, respectively) on PCoA coordinates to determine whether the given factors have a significant relationship with microbial community composition in the context of dietary interventions (Lang et al., 2018). In one case, while providing visualization with PCoA and statistical confirmation with ANOSIM, Vangay et al. (2018) additionally plotted ellipses for visualizing cluster centers/spread in their PCoA coordinates (Vangay et al., 2018). In another instance, Metcalf et al. (2017) showed the correspondence of dissimilarities between the 16S rRNA profiles and chloroplast marker profiles by performing a Procrustes analysis on the separate ordinations of the different data types (Metcalf et al., 2017).

We note that the choice of dissimilarity metric can have a significant impact on the low-rank embedding depending on the dataset. Shi et al. (2022) review the effect of high and low-abundance operational taxonomic units have on unsupervised clustering of Bray-Curtis and unweighted UniFrac (Shi et al., 2022). Marshall et al. (2019) compare Bray-Curtis ordination with weighted UniFrac on marine sediment samples and note that the most relevant clustering variable differed depending on the dissimilarity used (Marshall et al., 2019). These results imply that interpretation of low-dimensional embeddings and the putative driving variables must be performed in the context of the choice of dissimilarity. Metrics such as Bray-Curtis and weighted UniFrac take into consideration the abundance of individual microbes in each sample which can be important for datasets with many rare taxa. In contrast, some dissimilarity metrics such as Jaccard and unweighted UniFrac are only defined on binarized data, which may mask this property. Furthermore, phylogenetic metrics such as the UniFrac suite of metrics are best when the evolutionary relationships among microbial features is of interest in the context of sample communities. These metrics may also be more appropriate than other methods for datasets with particularly high sparsity.

PCA is arguably the most widely used and popular form of dimensionality reduction, which does not allow generalized beta-diversity dissimilarities (e.g., PCoA or UMAP), but does allow for the direct interpretation of feature importances relative to sample separations in the ordination. However, due to compositionality and sparsity, PCA often leads to spurious results on microbiome data (Hamady and Knight, 2009; Morton et al., 2017). Aitchison PCA attempts to fix these issues by using log transformation, but imputation is required (because the log of zero is undefined). Therefore, (Martino et al., 2019) proposed the adoption of RPCA for dimensionality reduction. This method has been shown to discriminate between sample groups in a wide array of biological contexts, including fecal microbiota transplants (Goloshchapov et al., 2019), cancer (Bali et al., 2021), and HIV (Parbie et al., 2021). Moreover, the generalized version of this technique accounts for repeated measures, allowing for large improvements in the ability to discriminate subjects by phenotypes across time or space (Martino et al., 2021). This advantage has been crucial in the statistical analysis of complicated longitudinal experimental designs such as early infant development models (Song et al., 2021). Feature loadings from these PCA-based methods can be used to inform selection of microbial features for log-ratio analysis (Morton et al., 2019; Fedarko et al., 2020), leading to novel biomarker discovery.

For feature interpretation, CCA is the most commonly used CA-based method for analyzing high dimensional microbiome data, due to its ability to incorporate sample metadata into the low-rank embeddings. This strategy has shown success in differentiating clinical outcomes following stem cell transplantation (Ingham et al., 2019) as well as diarrhea status in children (Dinleyici et al., 2018). CCA has also shown success in projecting environmental samples into lower-dimensional space such as in rhizosphere microbial communities (Benitez et al., 2017; Pérez-Jaramillo et al., 2017), and aerosol samples (Souza et al., 2021). Another approach designed for microbial feature interpretation has been posed by (Xu et al., 2021), explicitly modeling the ZGP through a zero-inflation model. This method attempts to optimize a statistical model for jointly estimating the “true” zero-generating probability as well as the Poisson rate of each microbial count.

Of non-linear methods, nMDS has historically been more widely used in microbiome data analysis, in part because it can incorporate an arbitrary dissimilarity measure. Furthermore, since nMDS is a rank-based approach, it is less likely than linear methods to be highly influenced by outliers in beta-diversity dissimilarities. Recent uses have involved using nMDS to show differences in the gastric microbiome between samples from patients with gastric cancer cases against the control of gastric dyspepsia (recurrent indigestion without apparent cause) (Castaño-Rodríguez et al., 2017) and demonstrating differences in the gut microbiome based on diabetes status (Das et al., 2021). In both of these cases, the visual distinction between groups was supported by PERMANOVA.

Other non-linear methods have been increasingly used for analyzing other types of sequencing data, especially in the single-cell genomics field, but have not yet been widely deployed in the microbiome. The most popular of these methods for visualization, t-SNE and UMAP, are starting to see more use in the microbiome field. (Xu et al., 2020) developed a method to classify microbiome samples using t-SNE embeddings. We recently reviewed the usage and provided recommendations for implementing UMAP for microbiome data (Armstrong et al., 2021). UMAP with an input beta-diversity dissimilarity matrix can reveal biological signals that may be difficult to see with traditional methods such as PCoA.

### Artifacts and Cautionary Tales in Dimensionality Reduction

Dimensionality reduction is incredibly useful and has led to many interesting biological conclusions. However, when using dimensionality reduction techniques, one must be careful how results are interpreted. There are known examples of patterns that are induced by the properties of the data alone (rather than the relationships among specific samples or groups of samples), and others that are a product of the method itself. Here, we discuss several known issues, as well as insights into evaluating the degree to which an ordination represents the actual data.

One of the most well-known artifacts in microbial ecology is the horseshoe effect (Podani and Miklós, 2002), wherein the ordination has a curvilinear pattern along what otherwise appears to be a linear gradient. This pattern can occur when a variable, such as soil pH (Lauber et al., 2009) or length of time of corpse decay (Metcalf et al., 2016) corresponds with drastic changes in microbiome composition on a continuous scale. Since the characteristic “bend” in the horseshoe typically occurs along the second coordinate of a PCoA (Figure 2B), it can obfuscate additional gradients/associations along that axis. Recent research in the topic has also identified that indeed, it is unlikely the horseshoe appears from a real effect, and instead it is a product of the limitations of many dissimilarity metrics to capture distance along a gradient when no features are shared between many of the samples (i.e., saturation) (Morton et al., 2017), which can be an issue with many common metrics, such as Euclidean, Jaccard, and Bray-Curtis dissimilarities (Morton et al., 2017). As a result, a possible remedy for the artifact is to use a dissimilarity metric that considers the relationships between features, such that two samples that share no features do not necessarily have the same dissimilarity as two different samples that share no features, e. g, UniFrac or weighted UniFrac. If a change in metric does not resolve the issue, it may be possible to avoid the horseshoe artifact by using RPCA or a non-linear method (e.g., UMAP). “Spikes” are another artifact, more prevalent on cluster-structured data, where outliers dominate the embedding and it fails to separate into clusters in the visualization (Vázquez-Baeza et al., 2017). Spikes also appear to be mitigated with an appropriate choice in dissimilarity metric, such as UniFrac (Hamady and Knight, 2009). In both cases, since the issues are with representing the distances between distant or extreme samples, non-linear methods (such as UMAP or nMDS) that dampen the effect of outliers provide a potential workaround to reveal secondary gradients or the obfuscated cluster structures (Armstrong et al., 2021). Though it is possible that the benefits offered by non-linear methods for the horseshoe effect are limited by the aspect ratio of the gradient (Kohli et al., 2021), and potentially the parameters of the algorithms.

Dimensionality reduction is also commonly used in other bioinformatic disciplines. Particularly, single-cell transcriptomics has used dimensionality reduction prolifically, with many publications using PCA, t-SNE, or UMAP visualizations. Furthermore, single-cell RNA-seq data shares many properties with microbiome data, including sparsity/zero-inflation, sequencing depth differences, and even phylogenetic relationships (Lähnemann et al., 2020). This connection is further strengthened by the fact that researchers in both disciplines investigate similar types of questions, albeit with different underlying data. Microbiome researchers often ask whether there is a difference between different treatments or disease-statuses (David et al., 2013; Lloréns-Rico et al., 2021), and which microbes contribute to those differences (i.e., differential abundance analysis). Similarly, transcriptomics may investigate parallel scenarios (Ocasio et al., 2019; Taavitsainen et al., 2021), where the goal is to discover transcripts whose expression stratifies the desired groups (i.e., differential expression).

Despite these similarities, the most popular methods for dimensionality reduction in microbiome and single-cell publications differ significantly, with PCoA being more prevalent among microbiome publications, and t-SNE (or variants (Linderman et al., 2019)) and UMAP more prevalent in single-cell publications (Kobak and Berens, 2019). Given the similarities in hypotheses and the properties of the data, but use of different methods, it is reasonable to suppose that methods such as t-SNE and UMAP have potential utility in the microbiome. However, global distances are not necessarily preserved in these methods, therefore distances between different clusters should not be interpreted as demonstrating similarity or dissimilarity. Consequently, recent research concerning the representation of single-cell RNA-seq data should also be taken into account when applying these methods to microbiome data.

First, t-SNE and UMAP are fairly complex algorithms that have many hyperparameters that can be adjusted, so it is important to be able to evaluate the faithfulness of the embeddings they produce. The evaluation of dimensionality reduction has been performed with many different measures, each of which has its own characteristics. Some measures reward embeddings that adequately preserve the local-scale structures in the embedding but do not necessarily penalize inaccurate representations of large distances in the original high-dimensional data, like the KNN evaluation measure (Kobak and Berens, 2019), which takes the average accuracy of the k = 10 nearest neighbors in the reduced dimensions compared to the original space. Others, such as the correlation (either Pearson or Spearman) between distances in the original space and reduced dimensions have been used (Becht et al., 2019; Kobak and Berens, 2019; Kobak and Linderman, 2021). The correlation measure generalizes whether the two representations overall are similar, i.e. close points in the original space are close in the low-dimensional space, and similar for far points. However, high correlation does not guarantee that the fine-scale structures have been preserved. Additionally, measures that use sample metadata about known classes can be used, such as the KNC measure (Kobak and Berens, 2019), which measures whether the closest class/category centers to a given category are preserved in the embedding. KNC emphasizes the preservation of relationships between classes, but not necessarily structures within the classes or between distant classes. These measures have been used to evaluate the quality of several dimensionality reduction methods across a variety of parameter settings on complex datasets. Notably, Kobak and Berens (2019) demonstrated on several single-cell transcriptomics datasets, that t-SNE with the default value for “perplexity” performed well at representing the relationships between nearby points (KNN), but poorly at representing the large-scale patterns (KNC and correlation). However, when they increased the perplexity parameter, they achieved improved KNC and correlation at the expense of a decreased KNN score. Kobak and Linderman (2021) observed with correlation that the best method (between t-SNE and UMAP) can vary by dataset. So, in practice, it may be necessary to compare multiple dimensionality reduction methods (and parameter settings) on a dataset using the measure that best suits the question, e.g., use the correlation measure when seeking a visualization of earth microbiomes by environment to show which environments are similar to each other.

Furthermore, since UMAP and t-SNE are algorithms that require configurable (possibly random) initializations, particular attention has been paid to their reproducibility. A metric to evaluate reproducibility comes from (Becht et al., 2019), which measures the preservation of pairwise distances in the embeddings by comparing an embedding on a subset of the points to the location of those points in the embedding of the entire dataset. In its original application, the reproducibility measure was used to demonstrate UMAP providing more reproducible results than t-SNE and variants of t-SNE. However, (Kobak and Linderman, 2021) showed that with appropriate (spectral) initialization, t-SNE can perform just as well by this metric as UMAP. While reproducibility is important, this metric should be applied carefully, because it fails to account for rotations in the embedding. Another important concern related to reproducibility is whether even random noise will yield apparent clusters. This phenomenon has been observed with t-SNE (Wattenberg et al., 2016), and whether other dimensionality reduction techniques are also susceptible to this effect warrants further systematic investigation. However, because these benchmarks are all performed within transcriptomics, further validation is needed to determine whether the conclusions generalize to microbiome data. These measures provide a starting point for evaluating the application of non-linear dimensionality reduction techniques on microbiome data.

Finally, literature from mathematics and computer science that has not been as widely applied to dimensionality reduction in bioinformatics may also be relevant. Of particular interest is the study of distortion, which is applicable when the goal of the embedding is to preserve distances, like one might expect for an exploratory analysis. Similar to the previously described correlation measure, distortion measures summarize the extent to which the distances in high dimensions match the distances in low-dimensions, however, distortion is defined in terms of the expansions and contractions of distances between points. Furthermore, there are many ways to summarize the expansions and contractions, including the worst-case, average-case and local-case, which are all detailed more in (Vankadara and von Luxburg, 2018).

## Discussion

The above examples illustrate that dimensionality reduction is an extremely powerful technique that has enhanced a wide range of microbiome studies. However, with great power comes great responsibility. It is unlikely that any one method will excel at representing all datasets, so responsible users of dimensionality reduction should try out several techniques, ideally guided by characteristics of the data rather than as a fishing expedition to see whether any one of many techniques produce results that “look good” (which may even happen in random data for some techniques and parameters) or that fulfill pre-conceived hypotheses and biases. We need standard protocols and software interfaces for choosing the algorithm that suits your data best, rather than the algorithm that shows what you want to see if you squint at it correctly. Methods are needed both for diagnosing the issues that may be most prevalent in your data and affecting your representation, and for rationally choosing among different methods that could be applied to a given dataset. Developing these methods is a key priority for the field.

Dimensionality reduction for the purposes of visualization has somewhat different goals from dimensionality reduction for other purposes and developing a better appreciation of this distinction is important for practice in the field. The goal of dimensionality reduction for visualization is primarily for exploratory overview by human observers (do groups differ from one another, is there overall structure such as gradients in the data). As such, visualization is usually done with three dimensions (more can be examined through parallel plots), while the intrinsic dimensionality of the data may be higher. Visualization is typically only the first step in the data analysis pipeline, and is followed by downstream analysis, such as multivariate analysis/regression (PERMANOVA, ANOSIM, PERMDISP) either on the original distances or on a dimensionality-reduced version of the data (which can be higher than three dimensions). These results can also be used to motivate supervised differential abundance modeling, such as to determine which groups separate and then determine which microbes are driving these separations.

Dimensionality reduction is thus often an early step in a multi-step pipeline. What downstream analyses is dimensionality reduction a step towards, and how are these accomplished? Feature loadings (i.e. the importance of particular taxa or genes) can be interpreted using log ratios from tools such as DEICODE (Martino et al., 2019), which can then be visualized in Qurro (Fedarko et al., 2020). Classification can be accomplished using machine learning techniques such as random forests, allowing estimates of classifier accuracy and group stability, and also allowing tests of the reusability of these models, e.g. applying a model of human inflammatory bowel disease to dogs (Vázquez-Baeza et al., 2016) or models of aging between different human populations (Huang et al., 2020). A popular strategy is to use a lower-dimensional embedding for traditional statistical analysis, such as using PCA or PCoA coordinates as inputs for regression, classification, clustering, and other analyses. However, as we have seen, many dimensionality reduction methods induce various kinds of artifacts or distortions, and cannot generalize well beyond the data on which the model was initially optimized on, including PCoA, nMDS, RPCA/CTF, and UMAP/t-SNE. Consequently, analyses on these coordinates should be performed with caution. Furthermore, since the parameters and software versions used with these methods have the potential to be highly influential to their results, we recommend that these always be reported for dimensionality reduction methods.

Given the large number of publications that have used dimensionality reduction on microbiome data, we can start to draw conclusions about which dimensionality reduction strategies should be more widely used, and which less widely used. On larger, sparser, compositional datasets, we recommend against the use of conventional PCA, Bray-Curtis and Jaccard dissimilarities, and pseudocounts. Conventional PCA presents the clearest case of a method that should not be used on microbiome data due the sparsity and compositional nature of the data. UniFrac and weighted UniFrac are essentially phylogenetically informed versions of Jaccard and Bray-Curtis beta-diversity metrics respectively. Due to the current default generation of a phylogeny in most 16S and shotgun analyses, there is no reason not to use the phylogenetic counterparts, which have been shown to have better discriminatory power. Pseudocounts should not be used because the choice of pseudocount impacts the lower-dimensional embedding, and there is no clear method for determining which pseudocount value is best.

In contrast, CTF and non-linear methods should be used more in microbiome contexts. As the cost of acquiring microbiome data continues to decrease, experimental designs are getting increasingly complex, and include repeated measures, longitudinal studies, batch effects, etc. We therefore need methods that can determine which biological signals are relevant among all these confounding factors. Additionally, we are increasingly recognizing that many relationships between/among samples are non-linear. Using non-linear methods can potentially explain more of such datasets with fewer dimensions, although additional benchmarking is required to understand the performance of these methods.

Our analyses suggest some important gaps in the field that could be important areas for future development. There are no dimensionality reduction methods yet that are both able to incorporate phylogeny and are compositionally aware. Several methods, such as Robust PCA and CTF, control for the sparsity, non-normality, compositionality, and are adaptable to specific study-designs of microbiome data but do not incorporate phylogenetic information. In contrast, phylogenetic techniques do not account for sparsity and compositionality, and some also perform poorly with non-normality. A unified method that is appropriate for any microbiome study is therefore still in the future, despite many important recent advances. The ability to perform this task using a generalizable dissimilarity measure would be particularly useful, because it would allow for full utilization of PCoA and non-linear methods including nMDS and UMAP.

Taken together, we conclude that dimensionality reduction is a key part of many, if not most, of the highest-impact microbiome studies performed to date. We can expect this situation to continue into the future, especially as larger study designs and datasets continue to accumulate, and additional method development advances increase the speed and range of applicability of these techniques.
